# European LeukemiaNet-defined primary refractory acute myeloid leukemia: the value of allogeneic hematopoietic stem cell transplant and overall response

**DOI:** 10.1038/s41408-022-00606-8

**Published:** 2022-01-17

**Authors:** K. H. Begna, J. Kittur, N. Gangat, H. Alkhateeb, M. S. Patnaik, A. Al-Kali, M. A. Elliott, W. J. Hogan, M. R. Litzow, A. Pardanani, C. A. Hanson, R. P. Ketterling, A. Tefferi

**Affiliations:** 1 Division of Hematology and Department of Internal Medicine, Rochester, MN USA; 2 Division of Hematopathology, Department of Laboratory Medicine, Mayo Clinic, Rochester, MN USA; 3 Division of Cytogenetics, Department of Laboratory Medicine, Mayo Clinic, Rochester, MN USA

**Keywords:** Acute myeloid leukaemia, Risk factors

## Abstract

We sought to appraise the value of overall response and salvage chemotherapy, inclusive of allogeneic hematopoietic stem cell transplant (AHSCT), in primary refractory acute myeloid leukemia (prAML). For establishing consistency in clinical practice, the 2017 European LeukemiaNet (ELN) defines prAML as failure to attain CR after at least 2 courses of intensive induction chemotherapy. Among 60 consecutive patients (median age 63 years) correspondent with ELN-criteria for prAML, salvage was documented in 48 cases, 30/48 (63%) being administered intensive chemotherapy regimens and 2/48 consolidated with AHSCT as first line salvage. 13/48 (27%) attained response: CR, 7/13 (54%), CRi, 2/13 (15%), MLFS, 4/13 (31%). The CR/CRi rate was 9/48 (19%), with CR rate of 7/48 (15%). On univariate analysis, intermediate-risk karyotype was the only predictor of response (44% vs 17% in unfavorable karyotype; *P* = 0.04). Administration of any higher-dose (>1 g/m^2^) cytarabine intensive induction (*P* = 0.50), intensive salvage chemotherapy (*P* = 0.72), targeted salvage (FLT3 or IDH inhibitors) (*P* = 0.42), greater than 1 salvage regimen (*P* = 0.89), age < 60 years (*P* = 0.30), and de novo AML (*P* = 0.10) did not enhance response achievement, nor a survival advantage. AHSCT was performed in 12 patients with (*n* = 8) or without (*n* = 4) CR/CRi/MLFS. 1/2/5-year overall survival (OS) rates were 63%/38%/33% in patients who received AHSCT (*n* = 12) vs 27%/0%/0% i*n* those who achieved CR/CRi/MLFS but were not transplanted (*n* = 5), vs 14%/0%/0% who were neither transplanted nor achieved CR/CRi/MLFS (*n* = 43; *P* < 0.001); the median OS was 18.6, 12.6 and 5.6 months, respectively. Although CR/CRi/MLFS bridged to AHSCT (*n* = 8), appeared to manifest a longer median OS (20 months), vs (13.4 months) for those with no response consolidated with AHSCT (*n* = 4), the difference was not significant *P* = 0.47. We conclude AHSCT as indispensable for securing long-term survival in prAML (*p* = 0.03 on multivariate analysis), irrespective of response achievement.

## Introduction

The 2017 European LeukemiaNet (ELN) characterizes primary refractory acute myeloid leukemia (prAML) as the absence of complete remission with count recovery (CR), after at least 2 courses of intensive induction chemotherapy [1]. Current literature on prAML is confounded by non-uniform disease definitions and study populations inclusive of relapsed AML [2–7]. AHSCT is proclaimed the best and only curative treatment option [2–6]. In the absence of controlled evidence for guidance, current prAML treatment is focused on achieving CR or CR without count recovery (CRi) first, through administering a panoply of salvage regimens, possibly affecting transplant candidacy. A 2020 report from MD Anderson Cancer Center (MDACC), cited rates of CR/CRi for AML refractory to one course of intensive higher dose (>1 g/m^2^) induction, as 11%, 5%, and 2%, after treatment with one, two, and three salvage regimens, respectively [8]. In general, response in prAML has been influenced by younger age (<60 years), favorable karyotypes, de novo context, blast percentage prior to salvage, and intensive salvage regimens [9–11]. In the current retrospective case series of 60 consecutive patients with ELN-defined prAML, we clarify the value and futility of salvage chemotherapy; identifying AHSCT as invaluable for procuring long-term survival regardless of response category prior to transplant.

## Methods

After approval from our institutional review board, the Mayo Clinic, Rochester, MN, AML database (*n* = 1800; 2004–2021) was queried to identify 60 patients conforming to the ELN 2017 definition of prAML [1]. All patients displayed persistent leukemia with >5% bone marrow blasts after 2 courses of ELN-classified intensive induction chemotherapy. Supplementary Table 1 delineates these intensive chemotherapy induction regimens.

All 60 patients received a lower or higher dose (>1 g/m^2^) cytarabine-based intensive regimen, either for their first or second induction course (outlined in Supplementary Table 2). A summary of the 2 intensive induction courses, subdivided into lower or higher cytarabine-dose based inductions is as follows:

*n* = 33 (55%) received 7 + 3 followed by higher dose (≥1 g/m^2^) cytarabine induction

*n* = 19 (32%) had 2 courses of lower-cytarabine-dose inductions:

7 + 3 (*n* = 17) or CPX351 (*n* = 2) followed by 7 + 3 (*n* = 14), or 5 + 2 (*n* = 4) or CPX351 (*n* = 1)

*n* = 3 (5%) had 2 courses of only higher dose cytarabine-based inductions

*n* = 1 (2%) received higher dose cytarabine + clofarabine, followed by carboplatin + topotecan

*n* = 1 (2%) received etoposide + mitoxantrone, followed by higher dose cytarabine + clofarabine

*n* = 3 (5%) had 7 + 3 followed by non-cytarabine intensive induction.

BM remission assessment approximated 4–5 weeks post induction, with repeat BM biopsies as merited for an aplastic or hypoplastic marrow. Karyotype was classified following ELN guidelines, into favorable, intermediate, and unfavorable risk category [1]. Next-generation sequencing (NGS) was performed in 37 patients and positive molecular-mutation statuses, aside from FLT3-ITD, FLT3-TKD, NPM1, were reported (Table 1), some patients (*n* = 9/60) having multiple positive mutations (Tables 2 and 3). ELN-designated salvage regimens, illustrated in Supplementary Tables 1 and 2, comprised intensive, less intensive or investigational, and molecular-mutation targeted therapy (quizartinib, gilteritinib, enasidenib, pan-IDH inhibitors). Salvage therapy was documented in 48 patients, inclusive of *n* = 2 who were bridged directly to transplant, and all were evaluable for a response (Table 1).

**Table 1:** Clinical characteristics of 60 patients with European LeukemiaNet-defined primary refractory acute myeloid leukemia (AML), stratified by the achievement of response after documented salvage therapy.

Variables	All patients *n* = 60 (100%)	Patients receiving documented salvage (*n* = 48)			*P*-value A vs B
		Patients with response (CR/CRi/MLFS) (Group A) *n* = 13 (27%)	Response rate (%)	Patients without response (Group B) *n* = 35 (73%)	
Overall response category					
CR, *n* (%)		7 (54)	15%		
CRi, *n* (%)		2 (15)	4%		
MLFS, *n* (%)		4 (31)	8%		
Age in years, median (range)	63 (23–78)	58 (27–72)		63 (23–78)	0.48
Age ≥ 60 years, *n* (%)	38 (63)	6 (46)	21%	22 (63)	0.30
Males, *n* (%)	40 (67)	9 (69)	29%	22 (67)	0.87
AML type					
De novo (primary), *n* (%)	28 (48)	9 (69)	38%	15 (43)	0.10
Secondary, *n* (%)	31 (52)	4 (31)	17%	20 (57)	
FLT3/NPM1 distributions: *n* = evaluable	*n* = 35	*n* = 10		*n* = 19	0.26
FLT3-ITD and NPM1 negative, *n* (%)	23 (66)	6 (60)	33%	12 (63)	
FLT3-ITD negative NPM1 positive, *n* (%)	1 (3)	0 (0)	43%	0 (0)	
FLT3-ITD and NPM1 positive, *n* (%)	3 (9)	0 (0)		3 (16)	
FLT3-ITD positive and NPM1 negative, *n* (%)	7 (20)	3 (30)		4 (21)	
FLT3-TKD positive and NPM1 negative, *n* (%)	1 (3)	1 (10)		0 (0)	
Positive molecular mutations on NGS: *n* = evaluable	*n* = 37	*n* = 6		*n* = 31	
ASXL1, *n* (%)	1 (3)	1 (17)		–	
BCOR, *n* (%)	1 (3)			1 (3)	
CEBPA, *n* (%)—silent	1 (3)			1 (3)	
CSF3R, *n* (%)	1 (3)			1 (3)	
DNMT3A, *n* (%)	2 (6)			2 (6)	
GATA2, *n* (%)	1 (3)			1 (3)	
KRAS, *n* (%)	1 (3)			1 (3)	
NRAS, *n* (%)	1 (3)			1 (3)	
RUNX1, *n* (%)	1 (3)			–	
SETBP1, *n* (%)	1 (3)			1 (3)	
SF3B1, *n* (%)	2 (6)			1 (3)	
TET2, *n* (%)	1 (3)			1 (3)	
TP53, *n* (%)	4 (11)			4 (13)	
U2AF1, *n* (%)	1 (3)			–	
WT1, *n* (%)	1 (3)			–	
IDH2, *n* (%)	1 (3)	–		1 (3)	
ELN karyotype at diagnosis: *n* = evaluable	*n* = 59	*n* = 13		*n* = 34	
Intermediate, *n* (%)	24 (51)	8 (62)	44%	10 (29)	**0.04**
Unfavorable, *n* (%)	35 (59)	5 (38)	17%	24 (71)	
% BM blasts before salvage therapy: *n* = evaluable	*n* = 60	*n* = 13		*n* = 35	
Median (range)	23 (0–99)	25 (5–99)	N/A	21 (0–90)	0.66
Documented number of salvage regimens to response or death: *n* = evaluable	*n* = 48	*n* = 13	27%	*n* = 35	0.16
One, *n* (%)	26 (54)	7 (54)	35%	19 (54)	
Two, *n* (%)	17 (35)	6 (46)		11 (31)	
Three or more, *n* (%)	5 (10)	0 (0)		5 (14)	
Salvage intensity:					0.47
Intensive, *n* (%)	30 (63)	9 (69)	30%	21 (60)	
Less Intensive, *n* (%)	13 (27)	2 (15)	15%	11 (31)	
Targeted, *n* (%)	5 (10)	2 (15)	40%	3 (9)	
AHSCT, *n* (%)	12 (20)	8 (62)	N/A	4 (12)	**0.001**
Relapse after AHSCT, *n* (%)		*n* = 8 2 (25)	N/A	*n* = 4 3 (75)	0.09

*ELN* European LeukemiaNet, *AML* acute myelogenous leukemia, *BM* bone marrow, *CR/CRi* complete remission with (CR) or without (CRi) blood count recovery, *MLFS* morphologic leukemia-free state, *AHSCT* allogeneic hematopoietic stem cell transplant, *Secondary* prior chemotherapy or radiation therapy-related AML, AML arising from prior myeloproliferative neoplasm, myelodysplastic syndrome, or chronic myelomonocytic leukemia, *FLT3* FMS-like tyrosine kinase 3, *ITD* internal tandem duplication, *TKD* tyrosine kinase domain, *NPM1* Nucleophosmin 1 = included 1 patient with favorable cytogenetics, *N/A* not applicable.

Bold values indicate statistically significant values.

**Table 2:** Baseline cytogenetic and molecular features of 13 prAML patients who achieved CR/CRi/MLFS.

Response type	Cytogenetics	Molecular profile	Age (years)	Number of salvage regimens to response	SCT	Progression-free survival (months)	OS (months)	Alive
MLFS	46, XY [20]	FLT3-ITD positive NPM1 WT	32	One: Decitabine + Sorafenib	No	2.6 (relapsed)	11.3	No
MLFS	46, XX t(7;13)(q21.2,q12) [2]/46, XX	FLT3-ITD positive NPM1 WT	56	Two: Cycle 1 HIDAC, then Mylotarg	Yes	7.1	13.2	No
MLFS	Unfavorable 46, XY, der(1)t(1;3)(p36.1;q21) [20]	FLT3-ITD WT NPM1 WT	55	Two: Idarubicin+ Clofarabine, then Decitabine	Yes	13.2	25.4	No
MLFS	Unfavorable 45, XY, −7 [10]	KIT positive FLT3-ITD WT NPM1 WT	60	Two: Cycle 1 HIDAC, then Clofarabine	Yes	114	120	Yes
CRi	46, XY [20]	FLT3-ITD positive NPM1 WT	62	One: Gilteritinib	No	1.1	5.7	No
CRi	Unfavorable 45, XX, der(3;5)(q10;p10) [12]/46, sl, +8 [4]/46, sl, +22 [4]	FLT3-ITD WT NPM1 WT	66	One: NK cell infusion with IL-15	No	1 (relapsed)	12.6	No
CR	Unfavorable +7, del 13q, +mar [20]	FLT3-ITD WT NPM1 WT	64	One: Clofarabine + Cytarabine	Yes	48	51.8	Yes
CR	46, XY [20]	FLT3-ITD WT NPM1 WT	46	One: MEC	Yes	Relapsed 1.8 months after CR, 2nd relapse 2 months after SCT	7.8	No
CR	Unfavorable 6p+, 7p+, 21q+ [20]	Not done	72	One: MEC	No	Unknown whether relapsed	24.8	No
CR	46, XY, t(2;11)(p21;q23) [20]	FLT3-ITD WT NPM1 WT	68	One: Cycle 1 HIDAC	Yes	No	72.8	Yes
CR	46, XY [20]	FLT3-TKD positive NPM1 WT	27	Two: Gilteritinib, then Decitabine + Venetoclax	No	1.6 (recent response)	9.5	Yes
CR	Unfavorable 46, XX,-3, del(5)(q13q33), +8, der(12)add(12)(p11.2) add(12)(q24.1) [20]	Not done	56	Two: MEC, then S-HAM	Yes	8	11	No
CR	46, XX, inv(2)(p23q31) [20]	Not done	58	Two: Clofarabine + Cytarabine, then MEC	Yes	6.6 (relapsed)	14.8	No

*CR* complete remission, *CRi* CR with incomplete count recovery, *MLFS* morphologic leukemia-free stat.

**Table 3:** Molecular profiles and outcomes of 27/47 primary refractory AML patients who received supportive care or documented salvage *without* response.

Patient	Molecular mutations	ELN-defined unfavorable karyotype	Age at diagnosis (years)	SCT	Number of salvage regimens	Type of salvage to SCT or death	OS (months)
1	TET2, SF3B1 FLT3/NPM1 WT		69		None		4.2
2	RUNX1, U2AF1, ASXL1 FLT3/NPM1 WT		72		None		4.2
3	NPM1 positive FLT3 WT	Yes	64		None		4.7
4	FLT3/NPM1 WT		65		None		16.0
5	FLT3/NPM1 WT	Yes	52		Unknown		9.2
6	FLT3/NPM1 WT		63		Unknown		21.1
7	MPL, NRAS FLT3/NPM1 WT	Yes	41		1	Decitabine + Venetoclax	2.6
8	TP53 FLT3/NPM1 WT	Yes	64		1	Azacitidine	3.6
9	GATA2, TP53, DNMT3A FLT3/NPM1 WT	Yes	62	Yes	1	Iomab	4.4
10	TP53 gene deletion FLT3/NPM1 WT	Yes	59		1	Decitabine	4.8
11	FLT3/NPM1 WT	Yes	65		1	Azacitidine	7.2
12	FLT3/NPM1 WT	Yes	46		1	Azacitidine	7.7
13	FLT3-ITD positive NPM1 WT		65		1	Decitabine + Sorafenib	8.3
14	FLT3-ITD positive NPM1 WT		23		1	CLAG-M	3.9
15	*FLT3-ITD positive NPM1 WT*		*62*		*2*	*CLAG-M, then Quizartinib*	*9.6*
16	FLT3-ITD positive NPM1 positive		65		2	Clofarabine + Cytarabine, then Azacitidine + Sorafenib	22.4
17	KRAS FLT3/NPM1 WT		64		2	Decitabine, then Cytarabine (1 g/m^2^)	3.4
18	CEBPA silent JAK2 positive FLT3/NPM1 WT	Yes	58		2	Clofarabine + Cytarabine, then Azacitidine + Sonedegib	3.9
19	FLT3/NPM1 WT	Yes	61		2	CLAG-M, then Decitabine	4.8
20	FLT3/NPM1 WT	Yes	63		2	Carboplatin + Topotecan, then Mylotarg	5.1
21	FLT3-ITD positive NPM1 positive		58		2	MEC, then Clofarabine	5.2
22	TP53, SF3B1 FLT3/NPM1 WT	Yes	64	Yes	3	MEC, then Carboplatin + Topotecan, then Iomab	22.4
23	*DNMT3A, IDH2*		*65*		*3*	*Azacitidine + Sonedegib, then Enasidenib, then pan-IDH inhibitor*	*12.5*
24	*BCOR/CBF AML FLT3-ITD positive (later in course)*	*Yes*	*50*		*4*	*5* *+* *2, then 1 cycle of cytarabine (3* *g/m*^*2*^*), then Azacitidine, then Gilteritinib*	*9.1*
25	FLT3-ITD positive NPM1 WT		25		5	1 cycle of cytarabine (3 g/m^2^), then Carboplatin + Topotecan, then Clofarabine, then Lurbinectedin, then Decitabine	12.3
26	FLT3-ITD positive NPM1 positive (complicated by myeloid sarcoma of sacrum)	Favorable t(8:21)	44		5	2 cycles of cytarabine (3 g/m^2^), then 7 + 3, then 1 cycle cytarabine (3 g/m^2^), then Clofarabine, then Carboplatin + Topotecan	18.7
27	SETBP1, CSF3R FLT3/NPM1 WT		53	Yes	AHSCT	Direct transplant (BM blasts 5%)	87.5

Therapeutic response categories included CR, CRi, and morphologic leukemia-free state (MLFS) pre-transplant or in those not proceeding to transplant, assessed until death. CR entailed <5% bone marrow (BM) blasts, absence of blasts with Auer rods, absence of extramedullary disease, absolute neutrophil count (ANC) ≥ 1000/μl, and platelet count ≥100,000/μl. CRi required all criteria for CR, but with either ANC < 1000/μl or platelet count <100,000/μl (not both). MLFS indicated BM < 5%, absence of extramedullary disease, absence of blasts with Auer rods, no hematologic recovery required, and BM cellularity of at least 10% or enumeration of 200 cells. No response indicated the absence of CR, CRi, or MLFS pre-transplant or in those not bridged to transplant, until death. Among patients who achieved morphologic remission (<5% BM blasts), minimal residual disease (MRD) was assessed by multiparameter flow cytometry and all MRD positive patients (*n* = 6) had MRD greater than 0.1% (*n* = 3/6, had MRD ≥ 1%). Date of last follow-up or death was updated until April 2021. Progression-free survival was measured from the date of CR/CRi/MLFS to the date of relapse or date of death/last follow up. Overall survival (OS) indicated date of AML diagnosis to death (regardless of cause) or last follow up. Statistical analysis was with JMP Pro 16.0.0 (SAS Institute, Cary, NC, USA). Categorical variables were assessed by chi-squared; continuous variables with Wilcoxon rank-sum test. The Kaplan-Meier method computed OS, with Cox proportional hazard models estimating covariate impact upon survival. *P* < 0.05 was considered significant.

## Results

A total of 60 patients (median age 63 years, range 23–78; 67% males) were diagnosed with current (2017) ELN-defined prAML. 8/60 (13%) received supportive palliative care, while an additional 4 patients were lost to follow-up after 2 courses of intensive induction. Forty-eight patients received documented salvage therapy (inclusive of 2 patients bridged directly to transplant) and were stratified according to response: 13/48 (27%) attained response: (CR 7/13 (54%), CRi 2/13 (15%), or MLFS 4/13 (31%)) (group A); and 35/48 (73%) no response (group B) (Table 1). The true CR rate was 7/48 (15%). Only 1 patient displayed a favorable karyotype and did not achieve a response (group B). Table 2 highlights the baseline cytogenetics and molecular features of all 13 CR/CRi/MLFS patients. Table 3 in parallel illustrates 27 molecular profiles and outcomes of patients who did not achieve any response. Aside from FLT3/NPM1, TP53 was the most prevalent mutation and only seen in those without a response, 4/31 (13%): all 4 patients had FLT3 WT/ NPM1 WT (wild type) and unfavorable karyotypes (highlighted in blue in Table 3), while only 1/4 (25%) had de novo AML. *n* = 2/4 (50%) with TP53 mutations proceeded to transplant with active disease (BM blasts > 30%) after being on the investigational Iomab-B salvage trial. OS for TP53 mutations: less than 5 months for *n* = 3 (75%), each of these patients received only 1 salvage regimen (hypomethylating agent, *n* = 2; investigational Iomab-B, *n* = 1), whereas *n* = 1 (received 3 salvage regimens: 2 intensive, then Iomab-B) had OS 22.4 months with AHSCT.

Univariate analysis identified intermediate-risk karyotype (44% vs 17% for unfavorable risk; *P* = 0.04) as predicting the likelihood of response (Table 1). Higher dose cytarabine induction did not predict response (CR/CRi/MLFS) attainment (8/38, 21%) vs lower dose cytarabine intensive induction (5/22, 23%), *P* = 0.88. Similarly, the receipt of any targeted salvage therapy vs all other non-targeted salvage did not predict response: 2/5 (40%) vs 11/43 (26%), respectively (*P* = 0.51). FLT3/NPM1 WT status (*P* = 0.26), BM blast percent prior to salvage (0.66), intensive (*P* = 0.72) or targeted salvage chemotherapy (FLT3 or IDH inhibitors) (*P* = 0.42), greater than 1 salvage regimen (*P* = 0.89) [*P* values involving salvage chemotherapy reflect those excluding the 2 patients bridged directly to transplant], age < 60 years (*P* = 0.30), and de novo AML (*P* = 0.10) also did not predict response, nor a survival advantage.

Patients receiving only less intensive salvage (16/48, 33%) had unfit status or prolonged complications from chemotherapy-induced cytopenias, such as transfusion dependence and neutropenic infections. In excluding the 2 patients bridged directly to transplant, on univariate and multivariate, median survival after any intensive salvage chemotherapy was 8.5 months vs. 7.4 months for only less intensive/targeted therapy (*P* = 0.08). The majority with only less intensive/targeted therapy received only 1 salvage regimen 14/16 (88%) vs 10/30 (33%) having intensive salvage (*P* = 0.001) In comparison to patients administered intensive salvage, the distribution of intermediate vs. unfavorable cytogenetics (*P* = 0.47), FLT3/NPM1 status (*P* = 0.22), age ≥60 years (*P* = 0.31), primary vs. secondary AML at diagnosis (*P* = 0.14), % BM blasts prior to salvage (*P* = 0.24), and attainment of response CR/CRi/MLFS (*P* = 0.72) with greater than 1 salvage (*P* = 0.30).

MRD evaluation after response in this cohort was limited, due to earlier period of AML diagnoses when MRD statuses were not routinely performed. MRD was assessed in 7/13 (54%) patients who achieved either CR (*n* = 5)/CRi (*n* = 1) /MLFS (*n* = 1). Only 1 of these 7 patients was CR-MRD Negative and bridged to transplant, the remaining 6 were MRD Positive (*n* = 2 were bridged to transplant). Irrespective of bridging to AHSCT, the univariate analysis did not display a significant difference in median survival *P* = 0.26. Median survival of MRD Positive patients was 14.8 months, the median survival of the *n* = 1 MRD Negative patient has not yet been reached. Similarly, there was no difference in median overall survival between MRD Positive >1% (*n* = 3, median 15 months) or MRD Positive >0.1% and <1% (*n* = 3, median 18 months), *P* = 0.59; or progression-free survival (6.6 months for MRD Positive >1% vs 12 months for MRD Positive >0.1%), *P* = 0.87.

Progression-free survival after CR/CRi/MLFS (3–12 months and greater than 12 months), on univariate analysis: was determinant on FLT3 WT and NPM1 WT status (*P* = 0.09), aggressive salvage (*P* = 0.01), and SCT (*P* = 0.01). Intermediate-risk cytogenetics, de novo AML, MRD positivity, and receipt of targeted therapy were insignificant. Multivariate analysis identified SCT as the most robust predictor of an enriched and enhanced response (*P* = 0.02).

AHSCT was performed in 12 patients including 8 from group A and 4 from group B. 8/12 (67%) died with 6/8 (75%) expiring from non-relapse related complications, median 9 months (0.5–67 months) post-AHSCT. These non-relapse complications were: [*n* = 1 severe refractory GVHD (graft vs host disease) 6 months post-transplant; *n* = 1 severe thrombocytopenia and transfusion dependence, refractory to Rituxan and prednisone, 12 months post-transplant]. This patient had developed multilineage MDS-RARS (myelodysplastic syndrome with refractory anemia and ringed sideroblasts) 10 months post-transplant; *n* = 1 severe refractory GVHD causing multiorgan failure, primarily of the gastrointestinal tract and liver, and ultimately septic shock, 4 months post-transplant; *n* = 1 had relapsed AML 1 month post-AHSCT with 10% BM blasts, however, expired from severe acute GVHD causing gastrointestinal tract and renal failure, 2 months post-transplant. This patient had been transplanted after Iomab salvage with persistent active BM blast burden 40%; *n* = 1 had severe cytopenias and subsequent septic shock 2 weeks post-AHSCT (patient had been transplanted with an aplastic marrow); *n* = 1 relapsed with 50% BM blasts 6 months post-AHSCT, and after reinduction, died from complications of chemotherapy-induced refractory cytopenias, 2 months post-AML relapse.

Transplanted patients in groups A and B manifested similar age distribution (≥60 years, *n* = 3 in group A vs 3 in group B, *P* = 0.21) while de novo AML was overrepresented in group A (75% vs 0% in group B, *P* = 0.005). Nine patients had cytogenetics evaluated prior to AHSCT, and 8/9 (89%) were classified as being intermediate-risk. The relapse rate after attaining any response (CR, CRi, or MLFS) was 31% (4/13), *n* = 3 had MRD status checked and were MRD positive. MRD status was assessed in 3/8 (37.5%) patients who had achieved a response prior to AHSCT, and all had CR. 2/3 (66%) had CR-MRD positivity >1% on flow cytometry: [*n* = 1 with CR-MRD^+^ remains alive at the date of last follow up April 2021, OS 73 months, with no evidence of relapse; *n* = 1 with CR-MRD^+^ had OS 15 months, experiencing relapse 6 months post-AHSCT]. The remaining 1/3 (33%) had CR-MRD negativity on flow cytometry. This patient remains alive, OS 52 months, with no evidence of relapse. Nonetheless, MRD positivity pre-AHSCT did not impact relapse risk (*P* = 0.31) or OS (*P* = 0.48) post-AHSCT in this small sample size. Interestingly, survival after AHSCT was adversely affected by female gender (*n* = 3/12, *P* = 0.17) which approached significance on univariate and multivariate; response status, time to transplant, BM blasts prior to salvage, greater than one salvage regimen, intermediate cytogenetic risk, de novo AML context, and age ≥60 years, additionally were insignificant factors.

Four patients were bridged to AHSCT without any response achievement. 2/4 (50%) were transplanted directly (*n* = 1 had an aplastic marrow and died 15 days post-transplant from cytopenias and multiorgan failure; *n* = 1 had low burden 5% BM blasts and a persistent clonal abnormality of trisomy 8. This latter patient relapsed 79 months after AHSCT in the form of isolated central nervous system myeloid sarcoma and remains alive). The remaining 2/4 (50%) were transplanted with a BM blast burden greater than 30% after being randomized to the investigational Iomab-B (anti-CD45) trial. [*n* = 1 had a high BM blast burden of 80%, and after Iomab-B had persistent active disease with greater than 40% blasts (approximating 50% cytoreduction) prior to AHSCT. This patient relapsed 1 month after AHSCT; *n* = 1 had failed two aggressive chemotherapy salvage attempts and had a BM blast burden 40% before Iomab-B, reduced to 30% BM blasts pre-AHSCT. AML relapse was noted 3 months post-transplant].

**Fig. 1:**
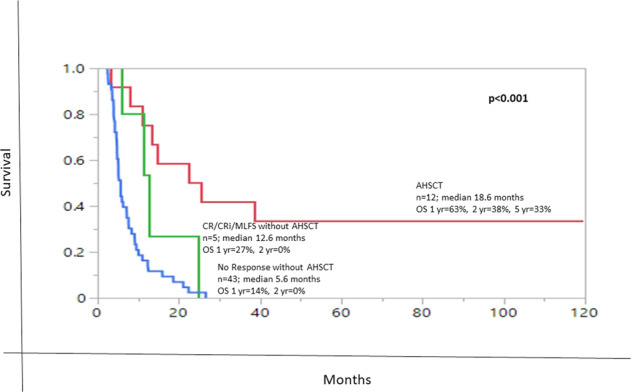
Overall survival (OS) stratified by AHSCT (allogeneic hematopoietic stem cell transplant).

**Fig. 2:**
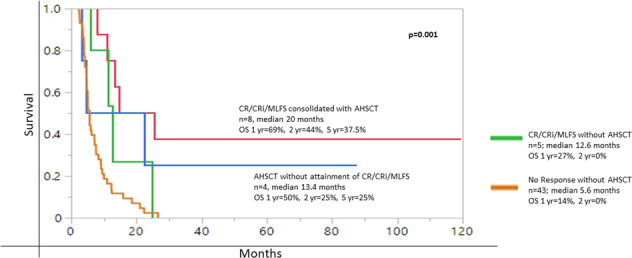
Overall survival (OS) stratified by AHSCT (allogeneic hematopoietic stem cell transplant) and response.

Median OS for all 60 study patients with prAML was 7 months (range 2.5–120 months); at last follow up, only 5 (8%) patients were alive, including 4/13 (31%) from group A and 1/35 (3%) from group B; 4/5 (80%) alive had been bridged to AHSCT. On univariate analysis, median OS after administration of intensive salvage chemotherapy (*n* = 30, 8.5 months vs *n* = 16, 7.4 months vs. *n* = 2 bridged directly to transplant, median not reached) (*P* = 0.15), achievement of CR, CRi, or MLFS (*n* = 7, median 25 months vs *n* = 2, 9 months, vs *n* = 4, 19 months vs no response, *n* = 47, 5.6 months) (*P* < 0.001), and bridging to AHSCT (*n* = 12, median 18.6 months vs *n* = 48, 5.7 months) (*P* < 0.001) prognosticated survival benefit (Fig. 1). Survival was not affected by higher dose cytarabine induction regimens (median survival 8.1 months vs 5.1 months for less intensive) (*P* = 0.69) or receipt of targeted salvage therapy (*P* = 0.93), (median survival 9.6 months (*n* = 5) vs 7.6 months (*n* = 43) with all other salvage therapy (intensive and less intensive/investigational)). Patients with CR/CRi/MLFS bridged to AHSCT (*n* = 8), appeared to manifest a longer median OS (20 months), vs (13.4 months) for those with no response consolidated with AHSCT (*n* = 4), *P* = 0.47 (Fig. 2). Multivariate analysis of the following predictive variables further confirmed AHSCT (Fig. 1) as the strongest predictor for long-term survival (*P* = 0.027), while achieving any response (*P* = 0.06) resulted in longer median survival, which was not durable in the absence of consolidation with AHSCT (Fig. 2); the type of response CR/CRi or MLFS vs no response (*P* = 0.88) became insignificant.

## Discussion

The current study addresses a frequent and practical question in the care of patients with prAML; is salvage chemotherapy and overall response worth pursuing, and what are the conditions for success or failure in that regard? Our observations suggest that salvage chemotherapy in prAML is clearly justified in the setting of consolidation with AHSCT. Although the number of informative cases was too small to allow definitive conclusions, we were encouraged that AHSCT retained its value in the absence of CR/CRi/MLFS, at the time of transplant. The latter point is noteworthy considering the limited merit of administering multiple salvage regimens for achieving CR/CRi/MLFS before transplant. For prAML patients who did not undergo AHSCT, the benefit from salvage chemotherapy was limited to prolongation of survival by a few months, and only in patients with intermediate-risk karyotype.

Consistent with existing literature on prAML, we confirm its dire prognosis with cited median survival ranging from 3 to 3.8 months in patients treated with supportive care alone [9] and 3–19 months for those receiving salvage therapy [9–14]. The ELN denotes regimens containing higher doses of cytarabine (≥1000 mg/m^2^) are generally considered the best option for those without response to a first course of 7 + 3. Achieving CR after a second course of a higher dose cytarabine regimen, may be lower in comparison to a second 7 + 3 induction after the failure of the first [1]. In this study, neither response (*P* = 0.88) nor survival benefit (*P* = 0.69) was seen with a higher cytarabine-dose induction compared to a lower intensive cytarabine induction. Of the 38/60 patients receiving higher-dose cytarabine induction either during their first or second course, 3/38 (8%) had been induced with 2 consecutive courses of only higher-dose cytarabine regimens. These 3 patients manifested a median survival of 2.6 months and failed to achieve a response, underlining the futility of higher dose cytarabine at the expense of efficacy and morbidity [1].

We also observed similar response rates and outcomes between intensive vs alternative modes of salvage chemotherapy. This is parallel to a retrospective study from MDACC where salvage regimens (intense vs HMA), not inclusive of transplant strategies, did not affect survival in 197 patients refractory to 1 course of high dose cytarabine induction [8].

AHSCT has been proposed as the only line of salvage that secures long-term survival in prAML with estimated 5-year survival rates of 20–30% [15, 16]. In a 2016 retrospective study of 8907 patients with newly diagnosed AML [17], 473 (5%) were identified as being refractory to 2 courses of intensive induction therapy, and their 5-year survival was reported at 8% vs 9% in patients refractory to 1 course of intensive induction vs 40% in those who achieved CR after 1 course of intensive induction; 86 (18%) of the 473 prAML patients proceeded to AHSCT and survival was superior in those who achieved CR after salvage chemotherapy (*n* = 49), compared to those transplanted in active disease (*n* = 37): 38% vs 17%, respectively. Though a small population 12/60 (20%), this is in contrast to our findings where CR/CRi/MLFS bridged to AHSCT (*n* = 8), appeared to manifest a longer median OS (20 months) vs (13.4 months) for those with no response consolidated with AHSCT (*n* = 4), the survival difference being insignificant, *P* = 0.47.

A 2020 study by Short et al. [18] at the MD Anderson Cancer Center focused on those undergoing AHSCT after response (CR/CRi/MLFS) with MRD negative or positive status. The cohort involved 141 patients with relapsed or refractory AML (88/141, 62% were refractory to induction or had remission lasting <1 year) in whom CR/CRi/MLFS was achieved after 1 cycle of salvage therapy and MRD status evaluated. Refractoriness was defined as refractory to 1 cycle of intensive chemotherapy or 2 cycles of less intense chemotherapy. 90 patients (64%) received intensive cytotoxic chemotherapy, and 51 patients (36%) had lower-intensity therapy (mainly HMA). Thirteen patients (9%) had undergone AHSCT. CR-MRD negativity was noted in 43% and manifested lower rates of early relapse, however, the 13 patients undergoing AHSCT (all in second remission) had the best outcomes despite MRD status or type of response. MRD status evaluation was limited in our cohort, with assessment in 7/13 (54%), and only *n* = 1 manifesting CR-MRD negativity, with a median survival not yet reached. The CR-MRD negative patient was bridged to SCT, and MRD positivity, >1% (*n* = 2) pre-AHSCT did not impact relapse risk (*P* = 0.31) or OS (*P* = 0.48) post-AHSCT. Though a limited sample, our findings corroborate with Short et al, in MRD status not affecting outcome post-SCT.

Limited clinical data exists on the significance of ELN-defined MLFS. Particularly, in our prAML cohort, outcomes were similar in MLFS vs. CR/CRi with or without transplant. This is consistent with a 2015 retrospective study on 270 AML patients, without primary induction failure, undergoing AHSCT [19], where 206 patients were in CR, 45 in CRi, and 19 in MLFS at time of transplant, with respective 3-year survival rates, post-transplant, of 49%, 46%, and 47% (*P* = 0.88). Survival after AHSCT, in prAML, requires further validation that should consider confounding effects from age, number of chemotherapy cycles before transplant, BM and peripheral blood blast %, and karyotype [20, 21].

The impact of intensive salvage chemotherapy for attaining CR/CRi prior to AHSCT has been studied in 845 AML cases failing one course of intensive induction chemotherapy [10], in which multivariate analysis revealed CR/CRi prior to transplant, predicted the best survival while older age and unfavorable karyotype adversely affected CR/CRi. Other reports have similarly addressed the importance of attaining CR before transplant [11]. On the other hand, AHSCT as first-line salvage, irrespective of achieving remission, has also been investigated in prAML [21–23]. In one retrospective cohort from MDACC involving patients refractory to 1 course of intensive induction, median survival was 16 months (3-year survival 39%) in those bridged directly to AHSCT (*n* = 28) vs 3 months (3-year survival 2%) with salvage alone (*n* = 149) (*P* < 0.001) [12].

Venetoclax (an oral highly selective Bcl-2 inhibitor) in combination with HMA, as opposed to venetoclax single-agent therapy, has shown promise in refractory/relapsed AML (defined as refractory or relapsed after one induction regimen of either intense or less intense (inclusive of HMA alone), or relapse post-AHSCT) [24–26]. Most patients received at least ≥ 2 salvage regimens prior to initiation. Toxicities (particularly grade 3 and 4 cytopenias: neutropenia and thrombocytopenia) complicate its use in refractory or relapsed AML. Nonetheless, activity has been shown in FLT3, TP53 mutations, and unfavorable risk karyotypes. After a median of 1 to 2 cycles of HMA + Venetoclax, overall response rates (CR/CRi) have ranged from 46% (41/90), median OS (7.8 months for all patients and 16.6 months for CR/CRi) [24]; to 33% (14/42), median OS 3 months vs 15 months for CR/CRi [25]; to 42% (23/55), 62% (34/55), median OS 7.8 months with response, and duration of response 16.8 months [26]. In this study, 2 patients received HMA + Venetoclax as a first or second line of salvage. One patient with NRAS, MPL positivity, FLT3/NPM1 WT had received 2 courses of high-dose cytarabine inductions with residual BM blast burden 80% and dural myeloid sarcoma. 1 cycle of decitabine + venetoclax was started, though discontinued due to severe pancytopenias (OS 2.6 months). The second patient was FLT3-TKD positive, NPM1 WT, biallelic WT1, who was started on gilteritinib as first-line salvage, cycle 2 halted due to elevated liver enzymes. BM blast burden had reduced >50% (from 40% to 11%). 2 months after, with BM blasts rising to 60%, FISH positive for RUNX1 in 53% of nuclei and a cryptic 3:1 translocation, cycle 1 of decitabine + venetoclax was started. CR was achieved after 1 cycle of decitabine + venetoclax with the disappearance of FLT3-TKD positivity and FISH abnormalities. MRD positivity on flow cytometry was observed to be 0.13%. The patient has continued with at least 3 cycles of decitabine + venetoclax, cycle 3 delayed due to a neutropenic PICC line infection.

In summary, our findings highlight the importance of AHSCT for securing long-term survival in a homogeneously exclusive cohort of ELN-defined prAML (primary induction failure), regardless of the degree of hematologic recovery. Currently, available salvage chemotherapy regimens (either intensive or less intensive (inclusive of targeted therapy)) for this prAML population were similar in regard to response or survival benefits. The promising clinical data acquired for HMA + venetoclax in combined refractory and relapsed AML requires further investigation in patients with primary induction failure and molecular mutations.

## Supplementary information

check list

Supplemental Table 1

Supplemental Table 2
