# Supplementary material for: European LeukemiaNet-defined primary refractory acute myeloid leukemia: the value of allogeneic hematopoietic stem cell transplant and overall response
Source: Blood Cancer J. 2022 Jan 17;12(1):7. doi: 10.1038/s41408-022-00606-8 (PMC8764050; doi:10.1038/s41408-022-00606-8)
Supplement: Supplementary file 2 — Supplemental Table 1 [file 41408_2022_606_MOESM2_ESM.docx]

**Supplementary Table 1:**

**European Leukemia Net-classified *Intensive* Induction or Salvage Chemotherapy Regimens Utilized in this Cohort**

| ***Cytarabine-based:***  “7+3”: 3 days of IV anthracycline: Daunorubicin at least 60 mg/m m^2^ or idarubicin 12 mg/m^2^ AND  7 days of continuous infusion cytarabine 100 to 200 mg/m^2^  “5+2”: 2 days of IV anthracycline: Daunorubicin at least 60 mg/m^2^ or idarubicin 12 mg/m^2^, AND  5 days of continuous infusion cytarabine 100 to 200 mg/m^2^  CPX351:  Encapsulation in nanoscale liposomes of a (5:1) molar ratio of cytarabine: daunorubicin  [daunorubicin 44mg/m^2^ and cytarabine 100 mg/m^2^, 90-minute infusion on days 1, 3, and 5].  ***Higher-dose Cytarabine (>1 g/m^2^) Regimens:***  MEC: mitoxantrone 8 mg/m^2^, etoposide 100 mg/m^2^, cytarabine 1 g/m^2^, days 1-5.  CLAG-M: cladribine 5 mg/m^2^ IV days 1-5, cytarabine 2 g/m^2^ IV days 1-5, filgrastim 300 mcg SQ days 1-5, mitoxantrone 10 mg/m^2^ IV days 1-3.  Clofarabine + Cladribine: clofarabine 40 mg/m^2^ as a 1-hour IV infusion followed 4 hours later  by cytarabine 1 g/m^2^/day every day for 5 days.  FLAG: Fludarabine 30 mg/m^2^/day IV days 1-5, followed 3.5 hours later by cytarabine 2 g/m^2^/day IV days 1-5, and filgrastim 400 mcg/m^2^/day IV until absolute neutrophil count (ANC) recovery.  HIDAC: Cytarabine 2000-3000 mg/m^2^ q12 hours IV, days 1-6 per each cycle  IDAC: Cytarabine 1000 mg/m^2^ q12 hours IV over 3 hours, days 1-3, WITH or WITHOUT  idarubicin 8 mg/m^2^ IV days 3-5, OR daunorubicin 45-60 mg/m^2^ IV days 1-3,  OR mitoxantrone 8 mg/m^2^ IV days 1-3.  Etoposide + Cytarabine: etoposide 100 mg/m^2^/day on days 1-5, and cytarabine 3 g/m^2^ twice daily on days 1-4.  SHAM: Cytarabine 3 g/m^2^ as a 3-hour continuous infusion, twice a day on days 1 to 2 and days 8 to 9 AND mitoxantrone 10 mg/m^2^, 30-minute infusion, days 3 to 4 and days 10 to 11.  ***Non-Cytarabine based***  Mitoxantrone + Etoposide: mitoxantrone 12 mg/m^2^/day and etoposide 100 mg/m^2^/day for 5 days.  Mylotarg (gemtuzumab ozogamicin): 3 mg/m^2^/day on days 1, 4, and 7 as monotherapy  Mylotarg + Mitoxantrone + Etoposide: mitoxantrone 10 mg/m^2^/day, days 1-3, etoposide 100 mg/m^2^/day over 2 hours, days 1-5, then Mylotarg 3 mg/m^2^ single infusion on day 6.  Carboplatin + Topotecan: carboplatin 150 mg/m^2^/day + topotecan 1.6 mg/m^2^/day for 5 days. |
| --- |
