# Supplementary material for: European LeukemiaNet-defined primary refractory acute myeloid leukemia: the value of allogeneic hematopoietic stem cell transplant and overall response
Source: Blood Cancer J. 2022 Jan 17;12(1):7. doi: 10.1038/s41408-022-00606-8 (PMC8764050; doi:10.1038/s41408-022-00606-8)
Supplement: Supplementary file 3 — Supplemental Table 2 [file 41408_2022_606_MOESM3_ESM.docx]

**Supplementary Table 2: *Less Intense* and *Targeted* Salvage Regimens Utilized in this Cohort**

| ***Less Intense:***  Nucleoside analogs  Clofarabine alone  Lurbinectedin (DNA-minor groove binder)-Investigational  Hypomethylating Agents (HMA)  Azacitidine  Decitabine  HMA + Multi-kinase inhibitor  Decitabine + Sorafenib  Azacitidine + Sorafenib  HMA + Hedgehog pathway inhibitor (anti-oncogenic proteins)  Azacitidine + Sonidegib (Hedgehog pathway inhibitor)-Investigational  HMA + Mitochondrial inhibitors (Bcl-2 inhibitor)  Decitabine + Venetoclax  ***Targeted FLT3-Inhibitors:***  Quizartinib  Gilteritinib  ***Targeted Epigenetic Inhibitors:***  Enasidenib  pan-IDH inhibitor  ***Investigational:***  ^131^I apamistamab (Iomab-B)-radio-immunotherapeutic  NK cell infusion with IL (interleukin)-15 |
| --- |
